# The mediation role of resilience and postpartum traumatic stress disorder on parental attachment and the maternal-infant bonding

**DOI:** 10.1186/s40359-023-01370-5

**Published:** 2023-10-27

**Authors:** Rong Nie, Mengxia Pan, Xinwen Liu

**Affiliations:** 1 College of Medicine and Health Science, Wuhan Polytechnic University, Wuhan, China; 2 Center for Reproductive Medicine, Department of Gynecology, Zhejiang Provincial People’s Hospital, (Affiliated People’s Hospital), Hangzhou Medical College, Hangzhou, Zhejiang China; 3 Department of Nursing, Zhejiang Provincial People’s Hospital, (Affiliated People’s Hospital), Hangzhou Medical College, Hangzhou, Zhejiang China; 4 Wuhan Children’s Hospital (Wuhan Maternal and Child Healthcare Hospital), Tongji Medical College, Huazhong University of Science & Technology, Wuhan, China

**Keywords:** Maternal-infant bonding, Parental attachment, Resilience, PTSD

## Abstract

**Aims:**

This study aimed to evaluate the correlation between parental attachment, resilience, postpartum traumatic stress disorder (PTSD), and maternal-infant bonding at 1 to 3 months postpartum. The mediation effect of resilience and PTSD on the postpartum parental attachment and maternal-infant bond was also evaluated.

**Design:**

A cross-sectional research design was used.

**Methods:**

A total of 400 postpartum women examined at a tertiary hospital in Wuhan from January 2021 to June 2021 were enrolled in the study. At about 1 to 3 months after giving birth, the women were asked to complete the Postpartum Bonding Questionnaire (PBQ), Connor-Davidson Resilience scale(CD-RISC), PTSD CheckList-Civilian version (PCL-C), and the Parental Bonding Instrument (PBI). The data were summarized using descriptive statistics. Mediation analyse and the Spearman correlation (r) were used to correlate the resilience and PTSD questionnaire scores.

**Results:**

The care attachment dimension was significantly associated with resilience (*r* = 0.24, *p* < 0.01), PTSD (*r* = − 0.27, *p* < 0.01), and maternal-infant bonding (*r* = 0.10, *p* < 0.01), and the overprotection attachment dimension was significantly associated with resilience (*r* = − 0.11, *p* < 0.01), PTSD (*r* = 0.33, *p* < 0.01), and maternal-infant bonding (*r* = 0.16, *p* < 0.01). Resilience and PTSD can mediate the relationship between attachment and maternal-infant bonding.

**Conclusion:**

Parental attachment, resilience, and PTSD significantly affect maternal-infant bonding at 1 to 3 months postpartum.

**Impact:**

This study demonstrated that new interventions aimed at addressing PTSD symptoms and improving resilience might increase parental attachment and maternal-infant bonding after birth. However, further research is required to evaluate the success of these interventions.

## No patient or public contribution

**Table:** 

Problem or Issue	Intergenerational transmission of maternal-infant bonding disorders and the occurrence of child abuse incidents frequently.
• What is Already Known	While a range of factors influence mater-infant bonding, women’s resilience and PTSD also play a role.
• What this Paper Adds	Parental attachment, resilience, and PTSD significantly affect maternal-infant bonding at 1 to 3 months postpartum, and resilience and PTSD play a mediation role in parental attachment and maternal-infant bonding.

## Introduction

Maternal-infant bonding is the process in which a mother forms an affectionate attachment to her infant [1]. This process starts during pregnancy and continues over the next few years. Maternal-infant bonding disorders (or failure) is a emotion disorder characterised by a lack of maternal emotional response towards her infant [2, 3]. Which can cause frequent crying and infant gastroesophageal reflux (GER), harm the maternal psychological well-being [4], poor emotional management skills within the newborn, and a serious long-term negative impact on the child’s development and maternal-infant interactions. Negative maternal-infant bonding will hinder infants’ regulation, tolerance, and integration of their own emotions and harm the infants’ social and emotional development, leading to the formation of poor emotional management ability [5, 6]. Studies have shown that postpartum maternal-infant bonding directly predicts social-emotional development in infants at 12 months of age [5]. About 5.2–11.3% of pregnant women suffer from a mild maternal-infant bonding disorder in the early postnatal period. Moreover, about 0.3–2.0% of mothers develop a severe maternal-infant bonding disorder and eventually reject their newborns [7, 8]. Several factors have been found to contribute to the development of maternal-infant bonding disorders, including the mother’s personality and educational level, spousal relationship, support from family and friends, postpartum anxiety and depression, and the newborn’s temperament [9, 10]. Early interventions may improve the maternal-infant bond and reduce the psychosocial burdens of the disorder. However, to develop effective interventions, more research is required to understand the processes involved in the development of maternal-infant bonding.

## Background

Parental attachment refers to the individual’s memories of the early years with their parents. Studies reported that a dual or disorganized attachment and anxious-ambivalent romantic, parental attachment styles tend to lead to increased bonding impairments [11, 12]. Protection and care are the main ways in which mothers care for their babies. In the attachment theory, parental attachment and maternal-infant bonding are intergenerational [13], meaning that the early attachment experience would create a prototype and provide a relationship engagement framework for later on in life [14]. As a result, numerous studies have found a positive association between parental attachment and maternal-infant bonding [11, 12]. More specifically, an insecure parental attachment was not only associated with the development of maternal-bonding difficulties [15–19], but also through the mediation effect of maternal sensitivity and emotional [20]. Further research is therefore required to understand the exact relationship between parental attachment and the onset of postnatal maternal-infant bonding.

Posttraumatic stress disorder (PTSD) is a delayed psychopathology stress disorder. Which can occur after childbirth trauma, affect about 3 to 4% of mothers, but the incidence can increase 4 to 5 times in patients with emergency deliveries, postpartum complications, and mental health issues [21, 22]. MacKinnon et al. [23] evaluated the impact of parental attachment on the development of PTSD at 5 weeks, 2 months, and 6 months postpartum and found that parental attachment was associated with the onset and rate of PTSD development. Furthermore, numerous studies found an association between PTSD and the development of maternal-infant bonding disorders [21, 24, 25]. On the other hand, Handelzalts et al. [26] found that avoidant and anxious maternal-infant attachments may occur due to PTSD symptoms.

Resilience is an ability in life that can enable individuals to cope with traumatic events and can also help individuals recover faster from adversity and stress [27]. As a result, several studies tried to evaluate the relationship between resilience and PTSD. Connor [27] developed the Connor-Davidson Resilience scale (CD-RISC) to measure resilience and found that individuals with higher CR-RISC scores could cope better with stress and were less likely to develop PTSD. The cluster analysis of Travis et al. [28] showed that women with poor infant attachment could enhance maternal-infant bonding by improving resilience. Resilience may have a mediation role in attachment and maternal-infant bonding. Similarly, other studies found that resilience may have an important role in preventing the intergenerational transmission of negative parental attachment feelings from the mother to the infant and thus enable the mother to cope better with stress [29, 30]. In addition, Wang Meifang et al. [31] identified resilience as an independent predictive factor for developing PTSD after 30 days to 1 year postpartum.

In the attachment theory, the relationship engagement framework and internal working models are thought to influence the perception of and coping with stressful experiences [14]. Hence, women with secure attachment experience may have a strong ability to cope with stress, actively adapt to childbirth events, and have a good maternal-infant bonding after birth. They have better resilience ability to overcome pressure, reduce the psychological or physical trauma or negative emotions caused by childbirth, and reduce the occurrence of PTSD [11, 14, 27].

Although numerous studies have evaluated the interrelationship between parental attachment, resilience, PTSD, and maternal-infant bonding, very few studies have evaluated the relationship between resilience and PTSD in mediating parental attachment and maternal-infant bonding. Therefore, further research is required to evaluate the role of resilience in reducing the symptoms of PTSD and hence enable healthcare professionals to develop interventions to enhance the maternal-infant bond and enable them to develop healthy parental attachments.

**Fig. 1:**
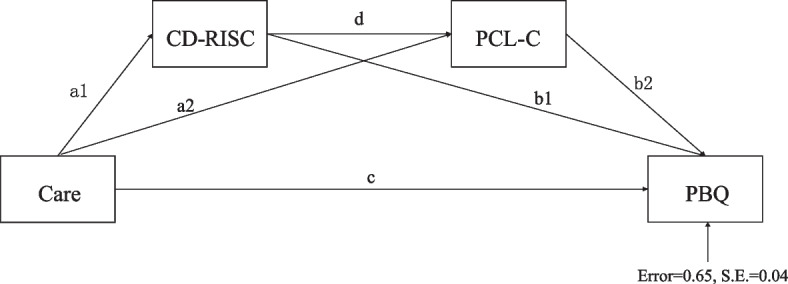
Note: a1: direct effect of parental attachment on resilience, a2: direct effect of parental attachment on PTSD, b1: direct effect of resilience on maternal-infant bonding, b2: direct effect of PTSD on maternal-infant bonding, c: direct effect of parental attachment on maternal-infant bonding, d: direct effect of resilience on PTSD. Mediation model of the effect of parental attachment on maternal-infant bonding by resilience and PTSD

Based on the above, Resilience and PTSD may affect the effect of parental attachment on maternal-infant bonding. Besides, we suspect that resilience and PTSD may play a role of chain mediation. The present study constructed a chain mediation model (Fig. 1) to test the mediating role of resilience, PTSD in parental attachment and maternal-infant bonding. We proposed the following hypothesis:

- Hypothesis 1: Parental attachment positively predicts maternal-infant bonding among maternal of 1 ~ 3 months postpartum.
- Hypothesis 2: Resilience plays a mediating role in the effect of parental attachment on maternal-infant bonding.
- Hypothesis 3: PTSD plays a mediating role in the effect of parental attachment on maternal-infant bonding.
- Hypothesis 4: Resilience and PTSD plays a chain mediating role in the effect of parental attachment on maternal-infant bonding.

## The study

### Aims

This study aimed to evaluate the correlation between parental attachment, resilience, PTSD, and maternal-infant bonding at 1 to 3 months postpartum. In addition, we also investigated whether resilience and PTSD can mediate parental attachment and the postpartum maternal-infant bonding.

### Design

A quantitative cross-sectional research design was used.

### Instrument 

The assessment tool consisted of 7 sections as follows.

#### Section 1: Socio-demographic and pregnancy childbirth-related information

This section aimed to collect information about the participants’ socio-demographic background (i.e., age and educational level), post-partum child and maternal health (i.e., days postpartum, parental responsibilities) and pregnancy and postpartum complications (e.g., hypertension during pregnancy, anemia, gestational diabetes, and sleep quality during pregnancy), and relationship status and satisfaction.

#### Section 2: Postpartum Bonding Questionnaire (PBQ)

In Sect. 2, the PBQ was used to measure postpartum maternal-infant bonding. The PBQ consists of 25 items divided into 4 dimensions: impaired bonding, rejection and anger, concerned with anxiety, and concerned with abuse. Each item is rated using a 6-point Likert scale, ranging from always to never [3]. The PBQ score ranges from 0 to 125, and a higher score indicates a worse maternal-infant bond. In the current study, Cronbach’s alpha was 0.88.

#### Section 3: The Connor-Davidson Resilience scale (CD-RISC)

In this section, the CD-RISC was used to measure the patient’s resilience. This questionnaire consists of 25 items, all of which are answered on a 5-point Likert scale ranging from (0) not true at all to (4) true nearly all the time [32]. The 25 items are divided into 3 dimensions: tenacity, strength, and optimism. The total score ranges from 0 to 100, with higher scores reflecting greater resilience. In the current study, Cronbach’s alpha was 0.87.

#### Section 4: PTSD check list-civilian version (PCL-C)

In this section, the PCL-C was used to measure the presence and severity of PTSD symptoms. PCL-C consists of 17 items, all of which are answered on a 5-point Likert scale ranging from (1) not at all to (5) extremely. The 17 items are divided into 3 dimensions: reexperiencing, avoidance/numbing, and hyperarousal. The total score of the questionnaire ranges from 17 to 85, and a higher score indicates higher levels of PTSD. The scale has good reliability and validity for measuring posttraumatic stress disorder in mothers [33]. In the current study, Cronbach’s alpha was 0.89.

#### Section 5: Parental bonding instrument (PBI)

The paternal bonding instrument was used to measure the contribution of parental behavior to the development of appropriate bonds between parents and children. The PBI has a version for the father and the mother. Each edition contains 25 items divided into 2 dimensions: care and overprotection. The Cronbach’s alpha of care dimension was 0.80, The Cronbach’s alpha of overprotection dimension was 0.86 in the current study. For this study, the version designed for the mother was adopted [34]. Each item in the PBI tool is rated using a 4-point Likert scale ranging from 0 (very unlikely) to 3 (very likely).

#### Section 6: Edinburgh postnatal depression scale (EPDS)

The Edinburgh Postnatal Depression Scale instrument was used to measure postpartum depression [35]. The EPDS consists of 10 items, all of which are answered on on a 4-point Likert scale ranging from (0) Nothing or very little to (3) always. The total score of the questionnaire ranges from 0 to 30, and a total score of more than 13 is classified as depression in this study [36]. In the current study, Cronbach’s alpha was 0.84.

#### Section 7: Self-rating anxiety scale (SAS)

The Self-rating Anxiety Scale instrument was used to measure postpartum anxiety [37]. The SAS consists of 20 items, all of which are answered on on a 4-point Likert scale ranging from (0) Nothing or very little to (3) always. Calculate the total score of 20 items, and the standard score is an integer after the total coarse score *1.25. A total score of more than 50 is classified as anxiety in this study [37]. In the current study, Cronbach’s alpha was 0.82.

### Sampling and recruitment

Convenience sampling was used to recruit the participants. Inclusion criteria:①≥18 years old; ②1 ~ 3 months postpartum and single live birth; ③Having the basic ability of reading and writing; ④Informed consent and voluntary participation. Women with a neurological or psychiatric disorder, serious pregnancy complications, a history of alcohol and drug abuse, and those with infectious diseases were excluded. In structural equation models, the minimum sample size is 10 times the number of items of the scale with the most items in the survey scale [38], The total of 25 items of the scale with the most items, so the sample size was expanded 10 times to 250, A total of 400 women participated in this survey.

The research team included trained obstetricians and gynecologists, pelvic floor rehabilitation specialists, and a graduate student. The researchers explained the purpose of the study, and written informed consent was obtained from all participants willing to participate. Another researcher explained the content of the questionnaire to those women who agreed to participate in the study.

### Data analysis

The Statistical Package for Social Sciences (SPSS) software version 23 and the Analysis of Moment Structures (AMOS) software version 24 were used for all statistical analyses. The continuous data were summarized as means +/- standard deviations (SD), and the categorical data were expressed as percentages. Spearman’s correlation analysis was used to test the correlation between the 4 variables.

The hypothesized mediation model was analysed using AMOS software (version 26). and AMOS 26.0 software was adopted to construct the mediating effect path analysis graph. In this study, well-established scales were used. Total scores of scales were used as observed variables, which simplified the analysis and interpretation of the data and mitigated the risk of model identification problems in the study with a small sample size. The level of significance was set at 0.05, care and over-protection are two dimensions of the PBI to measure parental attachment, that are negatively correlated and therefore analyze these two dimensions separately. Care and over-protection were two domains of the scale and were tested separately.

### Ethical consideration

The study was approved by the University Research Ethics Committee(BME-2021-1-10). Written informed consent was obtained from all participants in the study. Before completing the survey, the participants were informed that their participation in this study would not pose any harm and that all the provided information would be kept confidential.

## Results

### Characteristics of the participants

A summary of the characteristics of the participants is provided in Table 1. The mean age of the participants was 30.19 years, and most of the women (56.4) had an undergraduate degree or higher. 95% of participants reported symptoms of depression, and 25.2% reported anxiety symptoms. 70% of the participants had complications postpartum. With regards to sleep quality during pregnancy, 55.1% reported either good or very good sleep quality, and 80.5% were satisfied with their couple’s relationship. Depression (*p* = 0.01), anxiety (*p* < 0.01), complication (*p* < 0.01), couple relationship satisfaction (*p* < 0.01), and sleep quality(*p* < 0.01) had a significant impact on the PBQ score (Table 1).

**Table 1:** Characteristics of the participants (*N* = 401)

	Variable	N (%)/Mean (SD)	PBQ score	t/F	*p*
Age	Mother(years)	30.19 ± 3.59		0.87	0.73
	Infant(days)	52.2 ± 15.57		1.32	0.08
Education	Junior High School and below	25(6.2)	22.36 ± 10.71	2.17	0.09
	Senior High School	29(7.2)	15.66 ± 9.52		
	College	121(30.2)	20.17 ± 13.94		
	Undergraduate and above	226(56.4)	21.67 ± 12.52		
Who takes care of the baby	Totally self-care	39(9.7)	29.92 ± 12.73	1.76	0.84
	Care with others	359(89.6)	22.67 ± 6.11		
	Totally others-care	3(0.7)	19.77 ± 13.36		
parity	primipara	290(72.3)	21.15 ± 12.44	0.42	0.64
	multipara	111(27.7)	19.98 ± 13.53		
Depression	Yes	38(9.5)	25.71 ± 10.76	-2.50	0.01
	No	363(90.5)	20.31 ± 12.84		
Anxiety	Yes	101(25.2)	25.76 ± 12.46	-4.62	<0.001
	No	300(74.8)	19.16 ± 12.42		
Complications	Yes	120(29.9)	23.73 ± 13.92	-3.02	<0.001
	No	281(70.1)	19.58 ± 12.02		
Sleep quality	Very good	36(9.0)	15.33 ± 12.65	15.34	<0.001
	better	185(46.1)	17.65 ± 11.28		
	worse	159(39.7)	24.65 ± 12.03		
	Very bad	21(5.2)	29.19 ± 17.75		
Couple relationship satisfaction	Satisfactory	323(80.5)	19.55 ± 12.16	9.25	<0.001
	Fair	73(18.2)	20.40 ± 10.74		
	unsatisfactory	5(1.3)	26.51 ± 13.94		

*N* Number, *SD* Standard Deviation, *t/F* t/F -test; *p* *p*-Value;

### Association between attachment, resilience,PTSD, and maternal-infant bonding

Table 2 shows the mean ± SD score for each of the 5 variables and the Spearman’s correlation coefficient between each variable. The CD-RISC was positively correlated with the PBI care and negatively correlated with the PBI overprotection, PBQ, and PCL-C. The PBI care was negatively correlated with all other 3 variables. The PBI overprotection was moderately negatively correlated with the PBI care and CD-RISC. The PBQ was negatively correlated with CD-RIS and positively correlated with the PBI overprotection and care. The PCL-C was positively correlated with the PBQ and the PBI overprotection and negatively correlated with the PBI care and CD-RISC (Table 2). All correlations were statistically significant (*p* < 0.01).

**Table 2:** Descriptive statistics and Spearman’s correlation coefficient between the 5 study variables

	CD-RISC	PBI care	PBI overprotection	PBQ	PCL-C
CD-RISC	1				
PBI care	0.28^*^	1			
PBI overprotection	-0.29^*^	-0.55^*^	1		
PBQ	-0.37^*^	-0.31^*^	0.34^*^	1	
PCL-C	-0.25^*^	-0.32^*^	0.29^*^	0.54^*^	1

*Abbreviations*: *PCL-C *PTSD Check List - Civilian Version, *PBQ *Postpartum Bonding Questionnaire, *PBI *Parental Bonding Instrument, *CD-RISC *Connor Davidson Resilience scale

*; *p<*0.01

### Mediation analyses with resilience and PTSD as a mediator

**Fig. 2:**
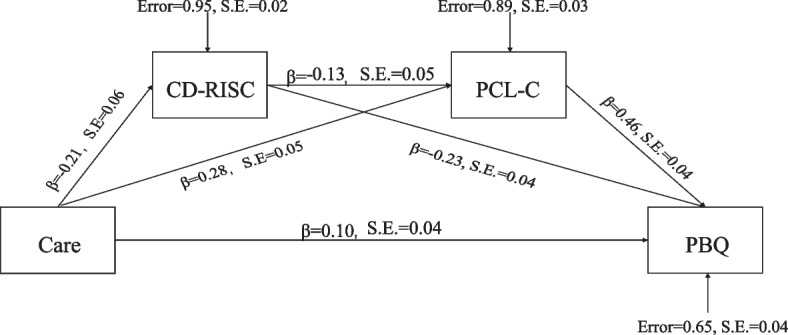
Note: a1: direct effect of care on CD-RISC, a2: direct effect of care on PCL-C, b1: direct effect of CD-RISC on PBQ, b2: direct effect of PCL-C on PBQ, c: direct effect of care on PBQ, d: direct effect of CD-RISC on PCL-C. *：p < 0.01. Mediation model of the effect of care dimension of attachment on maternal-infant bonding by resilience and PTSD

**Fig. 3:**
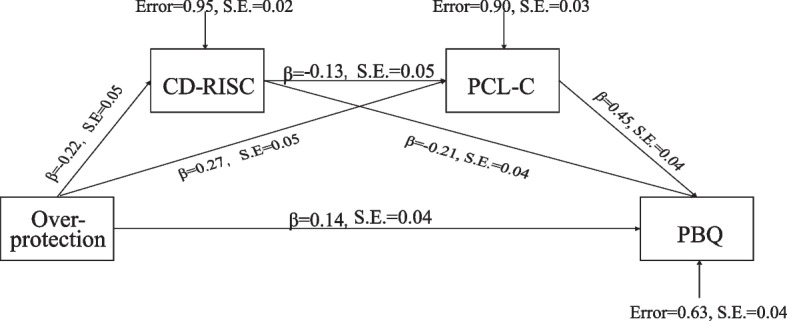
Note: a1: direct effect of Over-protection on CD-RISC, a2: direct effect of Over-protection on PCL-C, b1: direct effect of CD-RISC on PBQ, b2: direct effect of PCL-C on PBQ, c: direct effect of Over-protection on PBQ, d: direct effect of CD-RISC on PCL-C. *: p < 0.01. Mediation model of the effect of Over-protection dimension of attachment on maternal-infant bonding by resilience and PTSD

After controlling all the confounders, there was a significant sequential indirect effect of attachment on PBQ through CD-RISC and PCL-C. Figures 2 and 3 show all the possible pathways for the process model. All individual paths between the variables in the model were statistically significant.

In our study, the four variables of interest (independent variable, two mediators and dependent variable) were observed variables using corresponding total score. No latent variable is involved, so the model fit showed perfect performance with CFI=1, RMSEA=0, SRMR=0 and Chi-Square/df=0.

## Discussion

The present study investigated the relationship between attachment styles and maternal-infant bonding, in addition to exploring the mediating effects of resilience and PTSD. To our knowledge, this is the study to investigate the mediating role of resilience and PTSD in the relationship between parental attachment and maternal-infant bonding firstly. Our results show that the two dimensions of attachment (i.e., care and overprotection) relate to maternal-infant bonding at 1 to 3 months after birth. In addition, the relationship between attachment and bonding was mediated by resilience and PTSD.

Our findings also confirmed that parental attachment had both a direct and indirect effect on maternal-infant bonding. The direct effect can be explained by the feature of attachment and maternal-infant bonding, which are intergenerational in nature. [13]. Previous studies have shown that the dual-disorganized and anxious-ambivalent romantic attachment patterns had more bonding impairments [11, 12]. In our study, we observed that the care and overprotection dimensions can accurately predict maternal-infant bonding. Poor infantile care means a lack of emotional response in childhood, resulting in depression and other negative behaviors, which can be transformed into hostility, aggression, and other negative behaviors, which is not conducive to the development of positive psychology in children, and an overprotective maternal attachment also can increase the child's vulnerability risk factor and may prevent children from spontaneously developing their own potential [39, 40].

We also demonstrated that attachment results in maternal-infant bonding through resilience and PTSD indirectly. Resilience is viewed as the individual's capability to cope with stress, anxiety, depression, and major adversity. As a result, resilience may mediate the development of secure attachments. Secure attachments affect a person's emotional and psychological well-being. Stable emotions can lead to stable resilience when coping with traumatic events, making it less likely to develop PTSD and maternal-infant bonding [41]. As for the mediation of PTSD, it was shown that insecure attachment would compromise the mentalization of trauma and, therefore, children with insecure attachments are more likely to develop PTSD [42]. It is important to note that the main treatment for general PTSD is to avoid recalling the traumatic event. However, in cases of PTSD postpartum, it is not possible to remove the traumatic event. As a result, women may fail to develop a bond with their infant since the presence of the newborn can cause the mother to constantly recall the traumatic event of childbirth [43].

The findings of this study have a number of implications for clinical practice. In order to develop resilience, mindful-based stress reduction exercises could be used to enable the mother to focus on enhancing her ability to cope with stress [44] and improve their ability to cope with the life-changing impact of a newborn. A systematic review recommended several interventions to improve resilience, including enough rest and sleep, peer support, individual consultations, professional counseling, and information sessions [45]. However, the applicability of these interventions in addressing the targets identified in our study requires further research.

The standard treatment for women suffering from PTSD involves the use of psychotherapy and medications. However, the use of medications for the management of PTSD postpartum is generally avoided due to the low rate of adherence and the potential harm that these medications might pose to the child as a result of breastfeeding [46]. A meta-analysis concluded that psychotherapy in the form of cognitive behavioral therapy, reprocessing desensitization, and exposure therapy could reduce PTSD symptoms in the early postnatal period [47]. However, despite the potential benefits of psychotherapy in the management of PTSD symptoms, it is time-consuming and not widely available.

Nurses can provide psychological support to mothers by raising their own awareness on maternal-infant bonding disorders and the psychosocial interventions that could be used to support mothers. A nursing-led community postnatal follow-up service could also be used to identify women suffering from PTSD symptoms and bonding disorders at an early stage and hence provide timely interventions.

### Limitations

There are some limitations in this study. The maternal-infant bonding can change over time. However, since the questionnaires were administered only once, we could not capture the changes in maternal-infant bonding over time. Further longitudinal studies are therefore recommended. In this study, we only evaluated the impact of only 2 of the dimensions of attachment on the variables of interest. Future research could explore the impact of other attachment dimensions. In our study, we included all mothers irrespective of the health condition of their infant. Additional subgroup analysis could be performed to evaluate the impact of various conditions, such as premature birth and life-threatening health conditions, on the maternal-infant bond, resilience, and parental attachment.

## Conclusion

The present study suggests that resilience and PTSD can mediate parental attachment and maternal-infant bonding among women at 1 to 3 months after delivery. Therefore healthcare professionals should focus on developing psychosocial interventions aimed at addressing PTSD symptoms and improving resilience might increase parental attachment and maternal-infant bonding after birth. However, The effectiveness of interventions for mother-child relationships needs to be explored in the future.

## Data Availability

The datasets generated and/or analysed during the current study are not publicly available due [The work of this manuscript is a part of a research project which is currently not finished.] but are available from the corresponding author on reasonable request.
